# Cell death-based treatment of lung adenocarcinoma

**DOI:** 10.1038/s41419-017-0063-y

**Published:** 2018-01-25

**Authors:** Tatiana V. Denisenko, Inna N. Budkevich, Boris Zhivotovsky

**Affiliations:** 1 Faculty of Medicine, MV Lomonosov Moscow State University, 119991 Moscow, Russia; 2 Institute of Environmental Medicine, Division of Toxicology, Karolinska Institutet, Box 210, Stockholm, SE-171 77 Sweden

## Abstract

The most common type of lung cancer is adenocarcinoma (ADC), comprising around 40% of all lung cancer cases. In spite of achievements in understanding the pathogenesis of this disease and the development of new approaches in its treatment, unfortunately, lung ADC is still one of the most aggressive and rapidly fatal tumor types with overall survival less than 5 years. Lung ADC is often diagnosed at advanced stages involving disseminated metastatic tumors. This is particularly important for the successful development of new approaches in cancer therapy. The high resistance of lung ADC to conventional radiotherapies and chemotherapies represents a major challenge for treatment effectiveness. Here we discuss recent advances in understanding the molecular pathways driving tumor progression and related targeted therapies in lung ADCs. In addition, the cell death mechanisms induced by different treatment strategies and their contribution to therapy resistance are analyzed. The focus is on approaches to overcoming drug resistance in order to improve future treatment decisions.

## Facts

- Lung adenocarcinoma is one of the most aggressive and rapidly fatal tumor types.
- Resistance of lung adenocarcinomas to conventional radio- and chemotherapies represents a major challenge for treatment effectiveness.
- Combined therapies overcome resistance and are more effective than drugs targeting only one specific protein or pathway.

## Open questions

- What is the role of driving mutations in targeting therapy for lung adenocarcinoma?
- What should be done to improve the outcome of patients with tumors harboring specific alterations?
- Is crosstalk between different cell death modalities significant in combating lung adenocarcinoma?
- How can the resistance of lung adenocarcinoma to therapy be overcome?

## Introduction

Cancer comprises a highly heterogeneous and complex set of diseases associated with a variety of genetic and epigenetic aberrations. The “hallmarks of cancer” involve a set of cellular traits essential for malignant transformation and tumor maintenance. Among these are sustained proliferative signaling, induced angiogenesis, activation of invasion and metastasis, resistance to cell death, ability to escape immunological surveillance, and various others^1,2^. Genetic intra-tumor heterogeneity also can contribute to treatment failure and drug resistance. Despite extensive research, the intrinsic and acquired resistance of tumors to drug treatment remains a fundamental challenge in improving patient’ outcomes.

Lung cancer (LC) is the leading cause of cancer-related mortality^3^. Based on histology, LC is divided into two main subtypes: small cell lung carcinoma (SCLC) and non-small-cell lung carcinoma (NSCLC), accounting for 15 and 85% of all cases, respectively^4^. NSCLC is further classified into three types: squamous-cell carcinoma, adenocarcinoma, and large-cell carcinoma. Squamous-cell carcinoma comprises 25–30% of all LC cases. It arises from early versions of squamous cells in the airway epithelial cells in the bronchial tubes in the center of the lungs. The most common type of LC is adenocarcinoma (ADC), which comprises around 40% of all LC. Lung ADCs develop from small airway epithelial, type II alveolar cells, which secrete mucus and other substances^5,6^. Large-cell (undifferentiated) carcinoma accounts for 5–10% of LC. This type of carcinoma shows no evidence of squamous or glandular maturation and as a result is often diagnosed by default through the exclusion of other possibilities^7^. The discovery of mutated oncogenes, which encode activated signaling molecules that drive cellular proliferation and promote tumor growth, has now led to the development of more effective and less toxic targeted drugs for LC patients. However, similar to conventional chemotherapies, these new-targeted drugs also have a propensity to fail due to the development of resistance. Gene mutations and focal amplification are genetic changes that modulate the sensitivity of tumors to the induction of cell death, and, therefore, differences in treatment sensitivity may depend on the susceptibility of LC cells, in general, and lung ADC cells, in particular, to undergo cell death^8^.

Here we discuss recent advances in understanding the molecular pathways driving tumor progression and related targeted therapies in lung ADCs. In addition, the cell death mechanisms induced by different treatment strategies and their contribution to therapy resistance are analyzed. The focus is on the approaches to overcoming drug resistance in order to improve future treatment decisions.

## Driving mutations

Lung ADCs commonly contain a heterogeneous mixture of histological growth patterns, classified as “mixed type”^9^. Although histological features and marker expression remain the basis of clinical diagnosis, recent advances in sequencing technologies have led to an understanding of tumor heterogeneity and have allowed the further subdivision of lung ADC into molecular subsets according to a classification based on so-called driver mutations. These mutations represent molecular alterations essential for tumor initiation and growth. They can often be detected in genes that control cellular proliferation and survival^10,11^. Thus, tumors might rely on the expression of these single-mutant oncogenes to promote tumor growth and survival, also known as the concept of oncogene addiction^12,13^. As tumor cells depend on the aberrant activity of a specific mutated gene or pathway for survival and proliferation, their inactivation is generally sufficient to induce growth arrest and/or cell death^14^. An interesting hypothesis has been proposed to explain the phenomenon of oncogenic addiction. According to this hypothesis, the apoptotic response observed in tumors in the case of acute disruption of an oncogene product results from differential decay of several pro-survival and pro-apoptotic signals emanating from the oncoproteins^15^. The disturbance in the balance between pro-apoptotic and pro-survival signals could trigger oncogenic shock, which eventually might drive tumor cell death^15,16^.

**Fig. 1:**
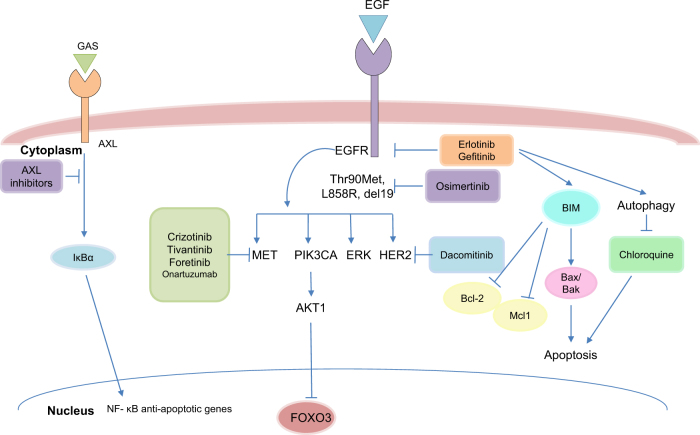
The EGFR-TKI resistance mechanisms and related targeted therapies in lung ADC The resistance to the EGFR-TKI involves activation of several pathways. One of the mechanisms depends on the appearance of secondary mutations in EGFR, such as Thr790Met, L858R or deletion of exon 19. Another one includes activations of bypass signaling pathways such as PI3CA, MET, ERK, HER2 or AXL. Low mRNA level or polymorphism of pro-apoptotic protein Bim could also mediate intrinsic resistance of lung ADC to EGFR inhibitors. Alternatively, autophagy stimulation might sustain resistance to the RTK inhibitors (erlotinib and gefitinib) in these tumors. The inhibition of autophagy with chloroquine could accelerate RTKI-induced apoptosis and overcome resistance of lung ADC cells. For more detail, see the text

The first actionable mutation detected in lung ADC was mutation in epidermal growth factor receptor (EGFR), a transmembrane receptor tyrosine kinase (RTK) that represents either somatic mutation deletion in exon 19 or L858R point mutation (Fig. 1)^15,17^. EGFR mutations near the ATP cleft of the tyrosine kinase (TK) domain result in increased receptor activation and act as oncogenic drivers. Binding with ligands (EGF and TGF-α) leads to conformational changes in EGFR and homodimerization or hetero-dimerization with other human epidermal growth factor receptor (HER) family members. There is subsequent auto-phosphorylation of the cytoplasmic TK domain with the help of adapter proteins (e.g., SHC and GRB-2), triggering downstream signaling pathways: (1) the rat sarcoma (RAS)/rapidly accelerated fibrosarcoma (RAF)/mitogen-activated protein kinase (MAPK) pathway; (2) the phosphatidylinositol-3-kinase (PI3K)/protein kinase B (AKT) pathway; (3) the Janus kinase (JAK)/signal transducers and activators of transcription (STAT) pathway. This stimulates mitosis, leading to cell proliferation and inhibition of apoptosis^18,19^. These pathways are crucial for normal cell growth. EGFR also serves as a stimulus for cancer growth.

Several types of activating mutations are known to occur in EGFR in NSCLC: Class I exon 19 in-frame deletions (44% of all EGFR mutations), Class II single amino acid changes (L858R 41%, G719 4%, other missense mutations 6%), and Class III exon 20 in-frame duplication/insertions (5%). All these mutations occur in the TK domain of EGFR. In 85% of all EGFR-activating mutations are exon 19 in-frame deletions or L858R, and they tend to be sensitive to currently approved EGFR inhibitors^20^. Class III mutations are generally insensitive to EGFR inhibitors with the exception of A763_Y764insFQEA^21,22^.

The next actionable genetic abnormality detected in NSCLC was the anaplastic lymphoma kinase (ALK) fusion oncogene, which is characterized by a fusion between echinoderm microtubule-associated protein-like 4 (EML4) and anaplastic lymphoma kinas (ALK). This fusion generates an overexpressed and activated TK, whereas normal lung tissue does not express ALK. EML4-ALK fusions are found in around 3–13% of lung ADC patients, and are largely mutually exclusive with alterations in other RTKs or KRAS based on analysis of almost 1700 tumors^23^.

KRAS is the most commonly mutated oncogene in lung ADC, with mutations detected in around 30% of patients^23^. Almost 97% of KRAS mutations in lung ADCs result in amino-acid substitution at codon 12 and 13. The mutated KRAS proteins exhibit impaired GTPase activity, resulting in constitutive activation of RAS signaling^24^. The presence of EGFR and KRAS mutations is also mutually exclusive in the same tumor. The role of KRAS mutational status as a marker of response to standard chemotherapy alone in NSCLC is poorly understood, but it has been clearly demonstrated that the occurrence of KRAS mutations is associated with the shortest survival of NSCLC patients treated with platinum-based and anti-EGFR therapies^25,26^.

Another targetable mutation in lung ADCs is c-MET, which also belongs to RTKs^27^. Binding with its ligand, hepatocyte growth factor (HGF), triggers receptor dimerization and phosphorylation, leading to conformational changes of c-MET that activate the TK domain as well as a wide range of different cellular signaling pathways, including those involved in proliferation, motility, migration, and invasion. Although c-MET is important for the control of tissue homeostasis under normal physiological conditions, it has also been found to be aberrantly activated in human cancers via gene mutation, amplification or protein overexpression^19^.

In addition, several other NSCLC driver mutations/gene translocations are currently under investigation, including ROS1/RET rearrangements, and BRAF/PIK3CA and HER2/MEK mutations, all of which might undergo specific targeted therapy^17^. However, it is remarkable, that in up to 40% of lung ADCs no driving mutations could be identified despite routinely used molecular diagnostics^28,29^.

### Targeted therapies

The identification of driver mutations in lung ADCs has led to the development of effective personalized treatment strategies (Table 1). Targeting the EGFR pathway represents a pioneering approach to personalized medicine in LC. Recently, a variety of TK inhibitors (TKIs) targeting EGFR have been tested in clinical trials and approved by the FDA^30,31^ First-generation EGFR TKIs, gefitinib and erlotinib, were designed to combine reversibly with the ATP-binding sites, thus blocking EGFR-induced activation of downstream signaling. The outcome of EGFR targeting is characterized by the disruption of a number of cellular processes that mirror the physiological consequences of EGFR signal transduction at the level of cell division, angiogenesis and apoptosis^32^. Different randomized controlled phase III trials have demonstrated that first or second generations EGFR TKIs represent the best first-line treatment option in patients with advanced lung ADC and whose tumors harbor EGFR mutations, considerably superior to conventional chemotherapy, because they significantly improved the response rate and progression-free survival (Fig. 1)^31,33–35^.

**Table 1:** Main genetic alterations in lung ADC and related targeted therapy

Gene	Gene alteration	Frequency, %	Targeted therapy	Potential targeted therapy	References
EGFR	Mutation or copy number gain	10	Erlotinib, gefitinib, afatinib and osimertinib	AZD9291, CO-1686, HM61713	Paez et al., 2004; Ji et al., 2006
ALK	Fusion	3–5	Crizotinib, ceritinib brigantinib	Alectinib, AP26113	Soda et al. ^53^; Chen et al., 2010
MET	Copy number gain	2–4	Crizotinib	Tivatinib	Engelman et al. ^79^; Xu et al., 2012
				Cabozantinib	
RET	Fusion	1	N/A	Carbozantinib	Kohno et al., 2012; Takeuchi et al., 2012
				Vandetanib, Alectinib	
ROS1	Fusion	1–2	Crizotinib	Cabozantinib	Rikova et al., 2007; Davies et al., 2012
KRAS	Mutation	15–25	N/A	Selumetinib plus docetaxel	Mascaux et al., 2005; Jackson et al., 2001
BRAF	Mutation	1–6	N/A	Vemurafenib, dabrafenib and trametinib	Paik et al., 2011;
PIK3CA	Mutation	5	N/A	BEZ235, BKM120 and GDC0941	Engelman et al., 2008; Kawano et al., 2006
MEK1	Mutation	1	N/A	Selumetinib and trametinib	Marks et al., 2008

As mentioned above, like EGFR mutations, ALK rearrangements define a unique molecular subset of NSCLCs. Crizotinib (Xalkori1; Pfizer, CA, USA) is a small-molecule ALK TKI that leads to cell arrest in the G1-S phase, and the induction of apoptosis^36^. Besides EML4-ALK, crizotinib is also active in tumors with c-MET gene amplification and c-ROS kinase mutations^21,37^. Crizotinib has now been approved by the FDA for the treatment of advanced, ALK rearranged NSCLC. The results from two early phase studies demonstrated impressive tumor response rates and prolonged patient survival^38^. Subsequently, several clinical trials, comparing crizotinib to standard chemotherapy in ALK-positive NSCLC patients showed significantly longer progression-free survival in the crizotinib group, leading to full approval of crizotinib by the FDA, EMA, and Japan^24,39^.

The outcome of TK targeting is characterized by the disruption of a number of cellular processes that mirror all levels of the physiological consequences of EGFR signal transduction^32^. Thus, in the context of oncogenic addiction, EGFR TKIs are able to influence the cellular level of apoptosis-related proteins underlying the pro-apoptotic effect of EGFR targeting^40,41^. It has been demonstrated that erlotinib-induced cell growth inhibition is accompanied by G1/S phase arrest, predominantly by the suppression of G1/S-related cyclins and upregulation of the CDK inhibitor p27KIP1^42^. Numerous studies have shown that the acute inactivation of EGFR results in a drastic decline in p-ERK and AKT and a delayed increase in p38 levels. This finally results in a rapid decrease in proliferative stimuli and an increase in pro-apoptotic signals in addicted cancer cells^43^. Orally administered gefitinib or erlotinib are taken up by cancer cells, and reversibly and competitively inhibit the binding of ATP to the phosphate-binding loop. Through the inhibition of ATP binding to EGFR, the EGFR TKIs block auto-phosphorylation and the activation of downstream signaling pathways, leading to the inhibition of cell proliferation and the induction of apoptosis in cancer cells^44^. Similarly, incubation with crizotinib results in a dose-dependent reduction in tumor cell growth, with a clear cell cycle arrest^45^. Inhibition of EML4-ALK with TAE684, a small-molecule ALK inhibitor, or via knockdown using RNA interference results in the abrogation of downstream signaling and induction of apoptosis through the activation of the pro-apoptotic protein Bim^46,47^.

Unfortunately, after TKI treatment, nearly all patients are eventually susceptible to disease progression due to acquired resistance (although resistant clones might have been present before treatment commenced)^22^. The resistant mechanisms identified can be categorized as secondary mutations in EGFR, bypass or alternative activations, or histological transformations^23,48,49^. The gatekeeper Thr790Met mutation is the most frequent secondary EGFR mutation, occurring in 50–65% of resistant re-biopsies. In addition, HER2 amplifications and mutations have been observed in lung ADC biopsies in 10 and 2% of tumors with acquired resistance to erlotinib and gefitinib, respectively, but in only 1% of untreated tumors^50^. Therefore, HER2 may also be responsible for resistance emerging under pressure of treatment, especially with erlotinib^51^. The development of third-generation irreversible inhibitor (AZD9291, osimertinib), which targets both Thr790Met and EGFR TKI-sensitizing mutations, has shown an objective response rate of 61% and a median progression-free survival of almost 10 months in patients with Thr790 Met-positive NSCLCs who progressed after previous TKI therapy (Fig. 1)^52^.

Secondary mutations in the ALK kinase domain have been identified in approximately 30% of ALK-positive patients with crizotinib resistance^53,54^. Acquired resistance to crizotonib in these patients inevitably leads to relapse and tumor progression. Moreover, the occurrence of de novo secondary ALK mutations results in variants that are intrinsically less sensitive to the drug^55^. Analysis of pleural fluid from these patients revealed two non-overlapping mutations, L1196M and C1156Y, within the ALK kinase domain. Each independently conferred crizotinib resistance *in vitro*. The L1196M substitution is notable because it involves the ALK gatekeeper residue, analogous to T790M in EGFR. The L1196M mutation, which replaces a leucine moiety with a bulkier methionine residue, likely causes resistance by steric interference with crizotinib binding. Since the initial case report of crizotinib resistance, additional second-site ALK mutations have been identified in patient-derived NSCLC specimens^54^.

To overcome these secondary mutations in ALK-harboring tumors, several novel ALK inhibitors have recently been developed. Agents, such as ceritinib, alectinib, and lorlatinib (PF-06463922, Pfizer; New York), have several potential advantages over crizotinib including better specificity (e.g., not inhibiting MET and ROS1), greater sensitivity, the ability to cross the blood–brain barrier, and different spectra of activity against resistance mutations to crizotinib^31,56,57^. All of these agents were initially assessed in patients with disease progression on crizotinib and showed substantial activity in this population with a response reported in at least 39% of patients and median progression-free survival of 5–7 months or more^58^.

Except for secondary mutations, in patients unaffected by TKI treatment wide ranges of resistance mechanisms have been reported. These include increased activity of additional kinases owing to MET, HER2 or ERK amplification, as well as an additional mutation of PIK3CA (which encodes the PI3K p110α subunit)^59^. Enhanced NF-κB signaling activity has also been proposed as one possible resistance mechanism that is evident from an improved response and survival in patients with EGFR mutations who have an increased expression of the NF-κB inhibitor IκBα (also known as NFKBIA)^60^. In addition, recent data have suggested the pro-apoptotic protein Bim as a biomarker and mediator of TKI-induced apoptosis in EGFR-mutated lung ADC^61^. The initial mechanism that might explain the different response of wt or mt EGFR lung ADCs to TKIs may include the EGF-mediated auto-phosphorylation of multiple tyrosine residues linked to the activation of distinct downstream effectors. These effectors may regulate level of Bim and Mcl-1 in mtEGFR cells but not in wtEGFR cells. Thus, the inhibition of EGFR in mtEGFR cells may initiate the apoptotic program via Bim/Mcl-1 alteration^62^. Moreover, the Bim polymorphism that results in changes in the splicing and deletion of the pro-apoptotic Bcl-2-homology domain (BH3) has been shown potentially to mediate intrinsic resistance to EGFR inhibitors, highlighting the complexity of possible resistance mechanisms (Fig. 1)^63^.

TKI resistance is also associated with epithelial-mesenchymal transition (EMT), a process characterized by a loss of polarity and cell–cell contacts in the epithelial cell layers, which undergo a dramatic remodeling of their cytoskeleton^64^. In the context of EMT, upregulation of the receptor protein TK AXL might lead to acquired EGFR TKI resistance in EGFR-mutant NSCLCs (Fig. 1)^65^. In addition, EMT regulated by loss of Mediator Complex Subunit 12 (MED12) has been shown to modulate the response to inhibitors of EGFR, ALK, and BRAF68 through negative regulation of TGF-βR2 leading to apoptosis^59^.

As tumors with KRAS mutations do not respond to either gefitinib or erlotinib, it has been suggested that the presence of these mutations be used as a biomarker for predicting resistance of lung ADCs to TKI therapy^25^. However, as described earlier, mutations in EGFR and KRAS are usually mutually exclusive, which makes KRAS an independent therapeutic target^66–68^. Mutations of KRAS are not a chemically druggable target but can potentially be treated with synthetic lethal approaches such as a combination of MEK inhibitors plus PIK3CA or AKT1 inhibitors^59^. For example, in second-line therapy, MEK has been targeted in KRAS-mutated NSCLC tumors by combining the MEK inhibitor selumetinib with docetaxel^69^. The suggestion to use MEK inhibitors in clinics was based on their ability to enhance apoptosis in KRAS and mutated NSCLC^70^. Unfortunately, despite the existing rationale for targeting downstream effectors of KRAS such as MEK, clinical trials have failed to confirm the efficacy of this strategy. A possible explanation, which has formed the basis of combined approaches, is an activation of compensatory signaling pathway(s) triggered by the inhibition of MEK, thus providing escape routes for the cancer cells that ensure their survival.

### Combination therapies

Although targeted drugs dramatically improve the outcome of patients with tumors harboring specific alterations, clinical responses are generally short-lived. In most patients with solid tumors, the cancer evolves to become resistant within a few months^34,71–73^. A possible approach to overcoming the limitations of targeted agents is to use combinations rather than monotherapies. For patients with acquired resistance, an option is to continue EGFR TKI therapy in combination with platinum-based doublet chemotherapy. This option is suggested to be beneficial because of the potential tumor heterogeneity at the time of EGFR TKI resistance. Early concurrent combination studies were designed before the discovery of EGFR mutations and the results of these studies in unselected populations showed that combination treatment did not improved survival compared with chemotherapy alone^74,75^. An explanation for this lack of efficacy is that the G1 cell cycle arrest caused by EGFR TKIs might reduce the cell cycle phase-dependent activity of chemotherapy^76^. In contrast, preclinical data have shown that sequential administration of TKIs after conventional chemotherapy might be effective^77,78^. Indeed, co-administration of chemotherapy with TKIs might attenuate the acute effect of EGFR withdrawal because of the effects of chemotherapeutic agents on DNA-damage checkpoints^14^.

As mentioned previously, resistance to TKIs may be developed through different mechanisms including upregulation of bypass signaling pathways. In this case the primary drug target remains unaltered and continues to be inhibited, whereas an alternative kinase becomes activated. There are multiple mechanisms of resistance via bypass pathways, such as MET or HER2 amplifications, and PIK3CA or BRAF mutations^23^. Amplification of MET, the main bypass signaling resistance, has been found in 20% of EGFR-driven resistant tumors, conferring resistance through ERBB3-mediated activation of downstream PI3K/AKT signaling, and effectively bypassing the inhibited EGFR^79^. Strategies aimed at co-targeting bypass pathways are being actively pursued in lung ADC^80^. There are several MET pathway inhibitors in clinical development, including TKIs (crizotinib, cabozantinib, tivantinib, and foretinib) and monoclonal antibodies directed against both MET (onartuzumab) and the HGF ligand (rilotumumab and ficlatuzumab)^80^.

HER2 has been found to be amplified in 12% of tumors with acquired resistance vs. only 1% of untreated lung ADCs. When amplified, HER2 is believed to function in parallel with the inhibited EGFR to reactivate common downstream signaling pathways^81^. Dacomitinib (PF-00299804, Pfizer; New London, CT, USA) is an irreversible pan-HER inhibitor that has shown remarkable activity in tumors with gefitinib-resistant EGFR T790M or HER2 mutations^79^. Recently, genetic alterations in effectors downstream of EGFR have also been identified as potential mediators of resistance^34,81^.

The ligand-independent activation of RTKs in lung ADC may arise from chromosomal rearrangements related to RTK genes and/or from point mutations or amplification of RTK genes^82^. The involvement of dysregulated RTK-dependent signaling in cellular transformation justifies the rational for the development of RTK inhibitors and their inclusion in targeted cancer therapy. However, the most recent RTK-targeted therapy failed to improve the cure rate because of the activation of defense mechanisms and acquired resistance in tumors^83^. One mechanism that might sustain the drug resistance of tumor cells is autophagy, which is known to be an important catabolic process that regulates the degradation and recycling of organelles and proteins within the cell, maintaining general cellular homeostasis^84^. Numerous scientific reports have highlighted the association between unbalanced regulation of autophagy and cancer^85,86^. Moreover, autophagy stimulation has been associated with resistance of lung ADC to RTK inhibitors, such as erlotinib or gefitinib^87^. Importantly, the degree of autophagy induction by erlotinib has been found to be greater in drug-resistant cells than in sensitive cells, suggesting that its induction may constitute a mechanism of cytoprotection^88^. The disruption of autophagy with chloroquine could accelerate erlotinib-induced apoptosis and overcome resistance of lung ADC cells to treatment with gefitinib or erlotinib. It has been reported that gefitinib causes a strong induction of autophagy in the NSCLC cell line PC-9^89^. The blockage of autophagic flux with clarithromycin was followed by marked induction of cell death^90^. ALK inhibition might also provoke autophagy-dependent resistance. In the same study, crizotinib, was used to generate resistant LC cell lines and the downregulation of ALK protein was shown to be associated with the induction of autophagy, demonstrating cytoprotective features^91^. Upon inhibition of autophagy with chloroquine, the sensitivity of drug-resistant LC cells to crizotinib was restored, providing an additional rationale for targeting autophagy in the case of resistance to RTK inhibitors^87,91^. The experimental evidence obtained after using MET inhibitors suggests that the cytoprotective effect gained by activating autophagy might be a serious obstacle to effective therapy. Therefore, the suppression of autophagy could be an approach to guaranteeing significant improvements in the therapeutic strategy of RTK inhibition^92^.

However, when autophagy is further elevated by treatment in addition to EGFR TKIs, it can induce autophagic cell death. Thus, in erlotinib-resistant HeLa-R30 cells, treatment with rapamycin increased autophagic cell death induced by erlotinib. Importantly, in this case cell death was inhibited by knockdown of the autophagy gene ATG7^93^. Similarly, in EGFR TKI-resistant LC cells with T790M mutation, the combination of a protein kinase CK2 inhibitor and an EGFR TKI led to a high level of autophagy that degraded EGFR protein and promoted apoptosis^94^. A recent study demonstrated that hypoxia-modulated autophagy was induced by EGFR TKI^95^. Furthermore, the pro-cell survival and pro-cell death roles of autophagy can be switched by adding EGFR TKIs early in hypoxia or by re-activating EGFR later in hypoxia^95^.

Interactions between malignant and neighboring nonmalignant cells create a dynamic tumor microenvironment that can also be therapeutically exploited. Important intercellular communications are driven by a complex and dynamic network of cytokines, chemokines, growth factors, and inflammatory and matrix remodeling enzymes against a background of major perturbations in the physical and chemical properties of lung tumors^96^. Angiogenesis is an essential process in the development, growth and metastasis of NSCLCs. Vascular endothelial growth factor (VEGF) is the major regulator of angiogenesis, and increased expression of VEGF is reportedly associated with poor prognosis^57^. A humanized antibody targeting VEGF, bevacizumab, has shown significant benefit when combined with cytotoxic chemotherapies^97,98^. Moreover, VEGF-targeted therapies exert their effects through a number of potential mechanisms, including inhibition of new vessel growth, regression of newly formed tumor vasculature, alteration of vascular function and tumor blood flow (“normalization”), and direct effects on tumor cells^99^. Because of the presumed cytostatic mechanism of action of anti-angiogenic agents, the efficacy of bevacizumab is most appropriately assessed through survival end points rather than the objective response end points that have traditionally been used with cytotoxic agents. VEGF mediates numerous pro-survival pathways in endothelial cells including induction or activation of Bcl-2, Akt, survivin and inhibitor of apoptosis proteins (IAPs)^100,101^. As VEGF mediates the survival functions of cancer cells, loss of VEGF signaling has been proposed to lead to cancer cell apoptosis^102^.

Recently, a phase II randomized study demonstrated that the addition of bevacizumab to erlotinib led to a significant benefit in terms of median progression-free survival in EGFR-positive NSCLC patients (16 vs. 9.7 months)^57^. Interestingly, the preliminary results of trial have revealed the benefit for patients with EGFR-T790M-positive tumors from treatment with the erlotinib plus bevacizumab combination with a median progression-free survival of 16 months compared to 10.5 months for patients without T790M, suggesting a new potential strategy for overcoming T790M-mediated acquired resistance^103^. However, bevacizumab has been shown to increase the response rate with chemotherapy in almost all tumor types studied in phase III trials. For safety reasons, these agents have been restricted to patients with lung ADC with a low risk of hemoptysis^104^. Patients with squamous cell morphology tumors were excluded from the trial because of an increased risk of bleeding events seen after bevacizumab treatment^105^. The FDA and EMA have approved a novel monoclonal antibody ramucirumab directed to the VEGF receptor for use in combination with docetaxel in the second-line treatment of squamous and non-squamous NSCLCs, although modest but statistically significant improvements in overall and median progression-free survival have been observed regardless of histological subtype^105^.

The largest class of anti-VEGF pathway agents comprises the TKIs that inhibit VEGFR. However, these compounds have multiple targets, leading to the variable toxicity and efficacy results seen to date. Nintedanib is an orally administered, small-molecule triple angiokinase inhibitor of VEGF1–3, PDGF-α and β, and FGFR1–3 which has demonstrated substantial antitumor and antiangiogenic activities in preclinical experiments and in clinical phase I/II trials in patients with NSCLC^106,107^. Nintedanib is the first antiangiogenic agent to demonstrate a survival benefit in the second-line treatment of patients with lung ADCs vs. docetaxel^108^. Further, two independent, multicenter, phase III studies assessed nintedanib combined with either docetaxel or pemetrexed in patients with advanced or recurrent NSCLC for whom first-line chemotherapy had failed. The combination of nintedanib and docetaxel significantly improved median progression-free survival vs. docetaxel alone regardless of histology, whereas overall survival was improved only in patients with lung ADC^108^. The combination of nintendanib and pemetrexed vs. pemetrexed alone in patients with non-squamous NSCLC significantly improved median progression-free survival but not overall survival^109^.

Sorafenib, a biaryl urea, is an oral small-molecule multikinase inhibitor that is effective against RAF kinase, VEGFR, platelet-derived growth factor receptor (PDGFR), c-KIT, c-RET, and FLT3 kinase^110^. This compound exhibit a significant broad-spectrum dose-dependent antitumor activity against a wide variety of human tumors in preclinical models. In addition to its anti-proliferative and anti-angiogenic effects, the anticancer effects of sorafenib are also thought to be mediated by apoptosis induction^110^. Recent studies have suggested that sorafenib-induced apoptosis is associated with downregulation of the antiapoptotic protein Mcl-1 and inhibition of eukaryotic translation initiation factor 4E (eIF4E) phosphorylation^111,112^.

Similar to sorafenib, sunitinib malate has been shown to be a potent inhibitor of VEGF receptors, FLT3, c-KIT, and PDGF receptors *in vitro* fulfilling its direct antitumor and anti-angiogenic properties^113^. Inhibition of VEGFR and PDGFR by sunitinib prevents further growth of new vessels^114^. Importantly, in other types of cancer one of the mechanisms for the partial resistance to sorafenib and sunitinib has been linked to authophagy development^87^. Moreover, the anticancer effect was restored when these drugs were combined with pharmacological inhibitors of autophagy (chloroquine or bafilomycin A1), which successfully re-activated apoptosis^115^.

Other agents that target VEGF directly, such as vandetanib, and cediranib, have been investigated for the treatment of NSCLC. However, despite promising results in preclinical studies, these compounds have not been found to provide clinical benefit (as measured by the median progression-free rate or overall survival) when combined with pemetrexed as a second-line treatment, compared with placebo, or with erlotinib in previously treated NSCLC patients^116,117^. Moreover, the clinical benefit of TKIs that inhibit VEGFR is limited by toxicity and acquired resistance.

Despite multiple resistance mechanisms and the complexities caused by tumor heterogeneity and microenvironment interactions, chemotherapeutics and molecularly targeted therapies are effective in many disease settings, significantly prolonging patients’ lives. The current challenge is to learn from experiences with traditional cytotoxic drugs and the first wave of molecularly targeted agents to use the increasing arsenal of anticancer therapies in the most effective way. Rational drug combinations are often proposed based on *in vitro* and *in vivo* synergy between agents. Most importantly, it is essential to stratify patients according to whether they are likely to respond to a particular drug or drug combination.

### Immunotherapy

LC initiation and progression depend not only on the evolving genomics and molecular properties of cancer cells but also on their interaction with the tumor environment, specifically with the immune system^118^. Although NSCLC has historically been considered a nonimmunogenic disease, emerging evidence has demonstrated that the lack of an effective immune response is in fact often the result of specific, active immune-evasive mechanisms, which, if understood, can be overcome therapeutically with significant clinical efficacy. Harnessing this potential has, therefore, become a primary area of clinical interest^119,120^.

The immune system is now recognized as having the potential to destroy cancer cells and inhibit tumor growth through the activation of innate and adaptive responses^121^. Innate immunity mediated by natural killer (NK) cells, polymorphonuclear leukocytes, and mast cells, as well as antigen-presenting cells (APCs), such as macrophages and dendritic cells (DCs), leads to the secretion of interferon gamma (IFN-γ) and perforin, as well as inflammatory cytokines that induce apoptosis of tumor cells. In contrast, adaptive immunity is controlled by T lymphocytes (CD4+ and CD+ cells) and antibody-producing B cells^121^. In this respect, adaptive rather than innate immunity offers the greatest potential for durable, robust anticancer immune responses. Of note, some of the cells involved in innate immunity, such as DCs, macrophages, and NK cells, also play a role in adaptive immunity^122^.

Interaction between the immune system and tumor is based on three phases: elimination, equilibrium and escape. Elimination is a phase of cancer immunoediting in which the innate and adaptive immune systems together detect and eradicate early tumor cells with activated cytotoxic T cells (CTLs) being major players. The first activating signal is associated with the interaction of T-cell receptors (TCRs) with major histocompatibility complex (MHC) class I molecules on antigen presenting cells (APCs), and provides specificity of response. The second, the so-called “costimulatory signal”, stimulates T cells after conjunction with antigen, and provides molecules on APCs that bind to particular costimulatory receptors on T cells. The best-known costimulatory molecules are the CD28 family^123^.

In the equilibrium phase the immune system holds the tumor in a state of functional dormancy. The escape phase is characterized by immunoediting insufficiency in restricting tumor growth^124^. As mentioned above, the ability of tumors to escape immunological surveillance is one of the hallmarks of cancer^1^. Entering the immune escape phase tumor cells are able to create an immunosuppressive state within the tumor microenvironment by subverting the same mechanisms that under normal conditions help regulate the immune response and prevent damage of healthy tissues^125^. Key immunosuppressive cell types found in the tumor microenvironment are regulatory T (Treg) cells, myeloid-derived suppressor cells (MDSCs), and tumor-associated macrophages^126^.

**Fig. 2:**
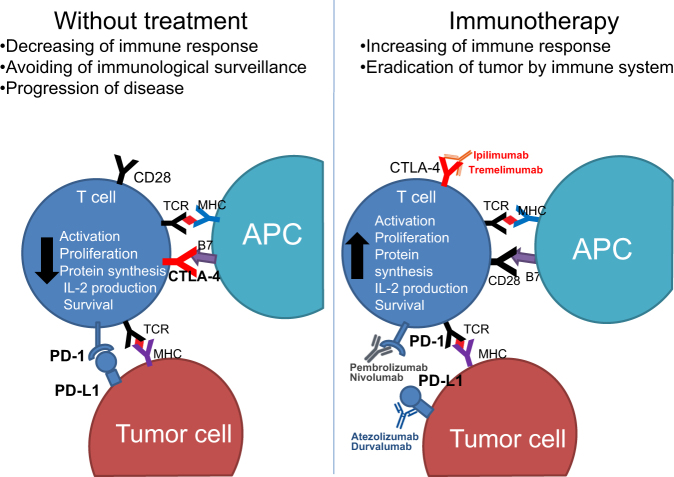
Link between the immune system state and response of immune checkpoint molecule inhibitors: CTLA-4 inhibitors (ipilimumab and tremelimumab), PD-1 inhibitors (pembrolizumab and nivolumab), PD-L1 inhibitors (atezolizumab and durvalumab) For more detail, see the text

Moreover, specific physiological regulatory mechanisms, or “checkpoints,” which play a key role in maintaining normal self-tolerance and limiting the extent of immune responses to infection, can be exploited by tumors as immune resistance mechanisms. Two of the checkpoint receptors most investigated in terms of immunotherapeutic targets for cancer are the cytotoxic T-lymphocyte antigen-4 (CTLA-4) and programmed death-1 (PD-1) receptor, which downregulate T-cell activation, proliferation, and function through different mechanisms (Fig. 2)^127^.

CTLA-4 is a protein receptor expressed on the surface of CTLs following their full activation. The binding between CTLA-4 and B7-1 (CD80) or B7-2 (CD86) on APCs prevents the hyperactivity of T cells under normal conditions. In tumors, T cells express a high level of CTLA-4, so that, cancer can evade the cytotoxic effect of T cells^128^.

Upregulation of the PD-1 receptor on activated T cells and subsequent binding to one of its ligands, programmed death ligand-1 (PD-L1) or PD-L2, provide an inhibitory signal during the effector phase of the T-cell response, reducing cytokine production, cell proliferation, and cell survival signaling^129^.

Like other tumor types, NSCLC can establish an immunosuppressive tumor microenvironment conducive to tumor growth^130,131^. For instance, NSCLCs have been shown to contain large numbers of Treg cells that constitutively express high level of CTLA-4 on their surface and directly inhibit T-cell proliferation^130^. In addition, in NSCLC, tumor-infiltrating CD8+T cells are characterized by increased PD-1 expression associated with impaired immune function. PD-L1 expression has also been found to be upregulated on NSCLC cells, shown correlation with the suppression of the maturation of tumor-infiltrating DCs and reduced tumor T-cell infiltration^131^. Furthermore, overexpression of PD-L1 and PD-L2 has been observed at more advanced disease stage in lung ADC^132^. Altogether, these data suggest the importance of immune checkpoints for targeting cancer cells. The administration of drugs targeting these molecular pathways may lead to complete or partial eradication of NSCLCs by the immune system.

Indeed, antibody-directed therapies against CTLA-4, PD-1, and PD-L1 have shown remarkable early success in the management of advanced NSCLC (Fig. 2)^133^. Several monoclonal antibodies directed to the PD-1 (nivolumab, pembrolizumab) or its ligand PD-L1 (atezolizumab, durvalumab, avelumab) are in clinical development, and nivolumab and pembroluzimab have been approved by the FDA and EMA for use in patients with advanced NSCLC who have previously been treated with chemotherapy (nivolumab has also been approved in Japan)^133^. Early clinical trials with these agents have shown rapid and durable responses in around 14–20% of previously treated patients with advanced NSCLC^134–136^. Even though progression-free survival has not been impressive (median 2–4 months), overall survival outcomes are remarkable^137^. Clinical efficacy seems to be independent of histology, but in most of the trials, greater benefit was seen in smokers and in patients with PD-L1-positive expression. The toxicity profile of these agents is quite favorable; however, some patients can respond with severe autoimmune disease.

### Combined therapy and immunotherapy

Currently, various combined immunotherapeutic regimens are being investigated in clinical trials for their ability to mediate superior antineoplastic effects compared to monotherapies^135^. Of interest is the combination of PD-1 blockade and CTLA-4 inhibition (nivolumab plus ipilimumab) and combination with other immune checkpoint modulators. In preclinical studies multiple immune checkpoint blockades with combination PD-1 and CTLA4 Ab treatment have been shown to allow increased T-cell responsiveness and decreased T-cell anergy^138^. These approaches have been supported by the results of clinical studies of nivolumab and ipilimumab in patients with NSCLC with squamous and non-squamous morphology^139^. However, a cautious approach is warranted given the potential to exacerbate autoimmunity.

Cytotoxic chemotherapy, targeted therapies and radiotherapy can modulate the immune response of tumors. An understanding of these immunomodulatory effects may enable the design of rational combinations of chemotherapy and immunotherapy. Several phase I/II clinical studies are currently under design to investigate PD-1/PD-L1 inhibition in combination with chemotherapy for patients with advanced NSCLC. Thus, nivolumab and atezolizumab in combination with chemotherapy as a first-line therapy have also shown promising clinical activity^140^. Cytotoxic chemotherapy with oxaliplatin, gemcitabine or paclitaxel can modulate the immune system through several mechanisms such as inducing immunogenic cell death, a form of cell death that forces DCs to stimulate tumor antigen presentation to T cells or stimulate T-cell activation via increasing the expression of MHC-1 molecules^141^. Moreover, these cytotoxic agents as well as cyclophosphamide may also stimulate DC maturation and reduce the immunosuppressive function of regulatory T cells^142,143^.

Combination therapy using PD-1 pathway blockade and EGFR TKIs has also been shown in preclinical studies to be promising. EGFR activation up-regulates the expression of PD-L1 and hence contributes to immune evasion^144^. A retrospective study that included 125 patients with NSCLC with mutant and wild-type EGFR, KRAS and ALK, all with PD-L1 expression revealed a correlation between PD-L1 expression and EGFR mutation^145^. Moreover, PD-L1- positive patients had higher sensitivity to EGFR-TKIs than PD-L1-negative patients in terms of the response rate. Interestingly, PD-L1-positive tumors were tightly linked to lung ADC histology^145^. A significant clinical benefit after TKI therapy in PD-L1-positive patients with EGFR-mutant advanced lung ADC was demonstrated^146^.

VEGF may have immunosuppressive effects via the stimulation of MDSCs in peripheral immune organs, promoting regulatory T cells and inhibiting DC maturation^147^. As such, VEGF inhibition in combination with a checkpoint inhibitor may have synergistic effects^148^. Several trials are currently assessing different aspects of the combination of antiangiogenic and immunotherapy, including a phase I trial evaluating the safety and tolerability of nivolumab as a maintenance therapy in combination with bevacizumab in NSCLC^149^.

### Adoptive immunotherapy

Other immunotherapeutic approaches including chimeric antigen receptor (CAR) and CD3-based bispecific agents have been associated with systemic cytokine release syndrome (CRS)^150^. CAR is a synthetic molecule designed to redirect T cells to specific antigens expressed on the surface of tumor cells^151^. CAR T cells can recognize antigens independently of human leukocyte antigen (HLA) unlike to the physiology of T cells and have the ability to affect tumor cells with low HLA expression or with the “wrong” antigen^152^. Tissue factor (TF) or coagulation factor III is overexpressed in many cancer types including LC^153^. The therapeutic efficacy of TF-CAR T cells has been estimated in a subcutaneous xenograft model in NOG mice using the human NSCLC cell line NCI-H292 containing the gene encoding luciferase (NCI-H292-luc). Intratumoral administration of TF-CAR T cells demonstrated significant inhibition of the growth of TF-positive NSCLC xenografts *in vivo*. In addition, TF-CAR T cells’ ability to suppress TF-positive NSCLC metastasis was revealed in a pulmonary metastasis model of the same mice^153^.

Recently EGFR has been evaluated as potent target for CAR T cell therapy, revealing a correlation between the infusion of CAR-T-EGFR cells and better response in treatment of 11 NSCLC cases^154^. Importantly, the CAR-T-EGFR protocol was safe and feasible for treating EGFR-positive advanced relapsed/refractory NSCLCs, suggesting that CAR T cell therapy could be a promising anticancer strategy for other solid tumors, particularly those with high EGFR expression.

An interesting approach in adoptive immunotherapy for NSCLCs is the use of T lymphocytes targeted to glypican-3 (gpc3). In terms of findings, firstly, immunohistochemistry assay showed that gpc3 was expressed in 66.3% of lung squamous cell cancer samples and in 3.3% of lung ADC samples but not in normal lung tissues. Second, in two established lung squamous cell cancer xenograft models, CARgpc3 T cells almost completely eliminated the growth of gpc3-positive cells. The ability of CARgpc3 T cells to persist *in vivo* and efficiently infiltrate the cancerous tissues was demonstrated. It seems that gpc3 might be a promising target for the treatment of squamous cell LC^155^.

## Conclusions

Unfortunately, lung ADC is still one of the most aggressive and rapidly fatal tumor types with overall survival less than 5 years. The discovery of oncogenic driver mutations and their role in predicting response to targeted therapies has changed the way in which clinicians approach the diagnosis and treatment of lung ADCs. Although targeted therapies have shown promising results, nearly all patients eventually have disease progression due to acquired resistance. In addition to well-known mechanisms, several novel mechanisms of resistance have recently been discovered, involving new resistance-conferring mutations within the target proteins (such as T790M in EGFR) or activating bypass signal-transduction pathways via unique mutations or changes in the expression level of the key proteins. During the past decade various new strategies aiming to induce cell death in lung ADCs and overcome their broad resistance to treatment, including acquired resistance to targeted therapies, have been developed. One of these is to combine traditional cytotoxic chemotherapy drugs with molecular-targeted compounds, which helps to overcome the limitations of targeted agents. Another approach to combating the resistance of lung ADC includes the combining autophagy inhibitors with RTK inhibitors. Accumulated evidence has revealed great promise in clinical trials using immune therapies for LC, in particular immune checkpoint blockade. The responses to this type of treatment tend to be durable, but still having problem related to reactions from patient to patient. Considerable effort should be made to understand the mechanisms that contribute to durable responses in some patients and lack of response in other patients with the same histological tumor subtype. It has become clear that personalized therapy is coming to the forefront cancer treatment. The selection of the proper therapeutic approach should be based on detailed analysis of histological features, the genetic (mutation) tumor profiles of individual patients, as well as the tumor microenvironment. This information will be essential for better prediction of malignant behavior and improvements in clinical management, which will include combination of targeted and immune therapies.
